# Biochemical Profile of an Adult Diabetic Population from Algeria in Relation with Anthropometric Parameters, Age and Gender

**Published:** 2018-08

**Authors:** Nour El Houda FERDI, Khalida ABLA, Haroun CHENCHOUNI

**Affiliations:** 1. Dept. of Applied Biology, Faculty of Exact Sciences and Natural Sciences and Life, University of Tebessa, 12002 Tebessa, Algeria; 2. Dept. of Natural and Life Sciences, Faculty of Exact Sciences and Natural Sciences and Life, University of Tebessa, 12002 Tebessa, Algeria; 3. Laboratory of Natural Resources and Management of Sensitive Environments ‘RNAMS’, University of Oum El Bouaghi, 04000 Oum El Bouaghi, Algeria

**Keywords:** Laboratory medicine, Type 1 diabetes mellitus, Type 2 diabetes mellitus, Body mass index

## Abstract

**Background:**

Diabetes is now a real global disease in particular due to various health problems associated with it. This study aimed to establish the relationship between diabetes and some biochemical parameters to assess the metabolic profile of an adult diabetic population in the region of Tebessa (Northeast Algeria).

**Methods:**

A cross-sectional survey was conducted at the Public Health Facility and the House of Diabetics in Tebessa between Feb 2013 and Apr 2013. The study included 200 subjects (100 controls and 100 diabetics) aged 18–85 yr, chosen completely randomly.

**Results:**

Type 2 diabetes mellitus (37%) was significantly more frequent than type 1 diabetes mellitus (13%). It was significantly more frequent in women than in men. The diabetes was highly correlated with the age and body mass index of patients. Moreover, the two types of diabetics have significantly more diseases and metabolic disorders compared to control subjects.

**Conclusion:**

Diabetics especially type 2 has significantly higher metabolic disorders and associated diseases than type 1 and control subjects.

## Introduction

In less than a quarter of a century, the diabetes mellitus became a major public health problem in developing countries (1,2). Nowadays, it is among the five main chronic diseases for which the WHO recently published a report calling for effective action (1).

Diabetes mellitus is a chronic non-communicable disease due to either genetic or acquired deficiency in insulin production (type 1 diabetes mellitus: T1DM), or a lack of action of this hormone (type 2 diabetes mellitus: T2DM). It is considered a serious public health because of its frequency, its social cost and its complications (3,4). Indeed, nearly 100 million cases of diabetes are reported and prevalent worldwide (5). “Different clinical forms of diabetes mellitus share hyperglycemia, which usually is chronic, and sometimes hyper-lipidemia and/or hyperproteinemia and consequently the ability to grow after a few yr of degenerative complications” (6). Indeed, diabetics generally are further affected by different diseases including obesity, hypertension, microangiopathy and metabolic syndrome (7,8).

In addition, T2DM is the more preponderant on the public health, particularly in Europe where 10 million people, the equivalent of one in 25 of the total population, are diabetics (9). Experts predict a global epidemic in the next 25 years, where they announced an alarming figure for the number of diabetics that could reach 300 million worldwide (9,10). Moreover, the growing epidemic of diabetes is more challenging in the developing world, particularly when it is associated with obesity (3,7). In Algeria, the prevalence of the disease is constantly increasing both in urban and rural populations (2). Indeed, the number of diabetics has increased from one million in 1993 to more than 2.5 million people in 2007, representing 10% of the national population in 2010 (11).

Globally, the risk of developing T1DM is low because it is actually influenced by inherited genetic factors. However, the risk of developing T2DM is higher and it increases with age, 20% of people over 65 yr suffer from T2DM (9, 12, 13). It is a multifactorial form. In addition to environmental and cultural factors, there is a strong genetic component (2, 11). Heredity, inbreeding, geographical and ethnic variations also play an important role. Factors such as body mass index (BMI), age group, reduced physical activity and dyslipidemia are also determinants (11).

Due to the important epidemiologic and economic consequences of the diabetes in the world in general and in developing countries in particular, this survey focuses on studying this health problem in the area of Tebessa (Northeast Algeria). The treatise represents a cross-sectional survey conducted in two hospital settings in Tebessa. It aimed to evaluate the metabolic profile of diabetic subjects with T1DM and T2DM in relation with their anthropometric parameters, age and gender; and to determine the impact of diabetes on the onset of other associated pathologies.

## Materials and Methods

### Data collection

The current prospective cross-sectional survey was conducted between Feb 2013 and Apr 2013 at the laboratory of Public Health Facility of Proximity (Department of Health) and the House of Diabetics in Tebessa. The survey included 200 subjects of both genders, including 100 diabetic patients (26 diabetic subjects with T1DM and 74 subjects with T2DM) and 100 control subjects randomly selected.

During the course of the investigation, age, gender, metabolic status (fasting glucose, total cholesterol, HDL, LDL, triglycerides, creatinine, and uric acid) and physical examination (weight, height, various pathologies associated with diabetes) were defined and/or performed for each study subject.

### Biochemical assay and anthropometric measurements

The biochemical parameters (glucose, triglycerides, total cholesterol, HDL, LDL, creatinine and uric acid) were measured on samples of blood using a digital mark BIOCHROMLIBRAS1 spectrophotometer. Then cholesterol ratio was calculated by dividing total cholesterol by HDL (14).

As for body weight, was determined using a portable scale of 150 kg range, to the nearest gram. Blood pressure was measured by a manual sphygmomanometer. The BMI was calculated based on simple measures of body weight (in kg) and height (in m): BMI = Weight/(Height)^2^.

The investigated subjects were classified into three groups based on BMI value: normal weight (BMI = 18.5–24.9 kg/m^2^), overweight subjects (BMI=25–29.9 kg/m^2^) and obese subjects (BMI ≥ 30 kg/m^2^). Then, obese subjects were grouped into three classes: Class I: moderate obesity (BMI = 30–34.9 kg/m^2^); Class II: severe obesity (BMI = 35–39.9 kg/m^2^); and Class III: massive or morbid obesity (BMI ≥ 40 kg/m^2^) (15). For each subject, all the data collected during an interview and the results of biochemical tests were registered on an individual record survey.

### Statistical analyses

All results were rounded into means with standard deviations (SD). The Student t-test was used to compare means of age, height, BMI and various biochemical parameters between diabetic subjects with two types with means of these parameters in controls. Comparison of percentages of sex, hypertension and microangiopathy between the two types of surveyed diabetic subjects was performed using the Chi-square test (*χ*^2^). The Pearson correlation test was used to identify the relationship between blood glucose, the parameter characteristic of diabetes, on the one hand and the various biochemical parameters and hypertension on the other hand. Statistical analyzes and tests of data were carried out at a significance level alpha = 0.05 using the software R (16) with the help of the package {Rcmdr}. Graphs were plotted using the package {ggplot2} (17).

### Ethical approval

All procedures performed in studies involving human participants were in accordance with the ethical standards of the institutional and/or National Research Committee and with the 1964 Helsinki declaration and its later amendments or comparable ethical standards.

Informed consent was obtained from all individual participants included in the study.

## Results

### Age and gender

The study population was characterized by an age ranging between 18 and 85 yr, with average of 47.44 ± 16.84 yr. The average age of controls, for a range of 18–72 yr, was significantly different (*P* = 0.001) than the mean age of diabetic subjects that ranged from 19 to 85 yr. Whether men or women, the diabetics were statistically significantly older. Moreover, subjects with T2DM were significantly older than patients with T1DM (Table 1).

**Table 1:** Distribution of the surveyed population according to age and gender

***Parameters***		***Men***	***Women***	***Both genders combined***
Control subjects	N (%)	38 (38)	62 (62)	100 (100)
	Age (years)	39.26 ± 15.22	40 ± 15.44	39.72 ± 15.29
T1DM	N (%)	07 (26.93)	19 (73.07)	26 (100)
	Age (years)	40.71 ± 21.24	48.16 ± 16.63	46.15 ± 17.85
	*P*	0.714	0.068	0.072
T2DM	N (%)	23 (31.08)	51 (68.92)	74 (100)
	Age (years)	59.35 ± 10.75	57.88 ± 12.63	58.34 ± 12.02
	*P*	<0.001	<0.001	<0.001
Both diabetes types	N (%)	30 (30.00)	70 (70.00)	100 (100)
	Age (years)	55.00 ± 15.66	55.24 ± 14.37	55.17 ± 14.69
	*P*	<0.001	<0.001	<0.001

(N: number of subjects, *P*: *P*-value of unpaired *t*-test between controls and diabetics)

Regarding gender, women were significantly (un-paired t-test: *P* = 0.004) more numerous in the two study groups (diabetics and controls). However, there were no significant differences between genders in control and diabetic subjects of both types (Table 1).

The study of the correlation indicated a statistically significant positive relationship between blood glucose and age of the control subjects (*r* = 0.154, *P* = 0.025), as well as for the age of patients with T1DM (*r* = 0.075, *P* = 0.046) and T2DM (*r* = 0.128, *P* = 0.006), regardless of gender.

### Anthropometric measurements

**Fig. 1:**
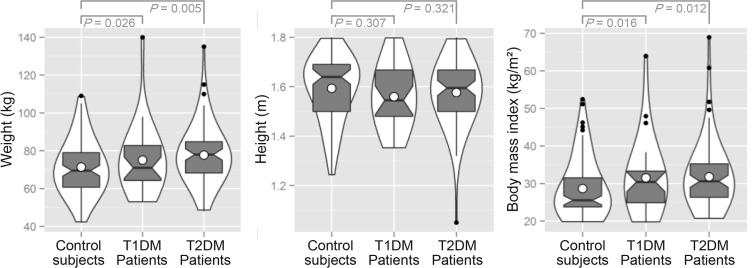
Violin plots with overlaid notched box plots representing the distribution of anthropometric parameters (weight, height, BMI) of the study population in Northeastern Algeria. The open circles are markers of the means, whereas black dots are box plot outliers. *P*: *P*-value obtained from unpaired *t*-test

The averages of BMI and weight in diabetics (T1DM and T2DM) were significantly higher compared to control subjects. While the controls were relatively taller than of diabetic subjects, but height was no significantly different for both types of diabetes (Fig. 1).

**Fig. 2:**
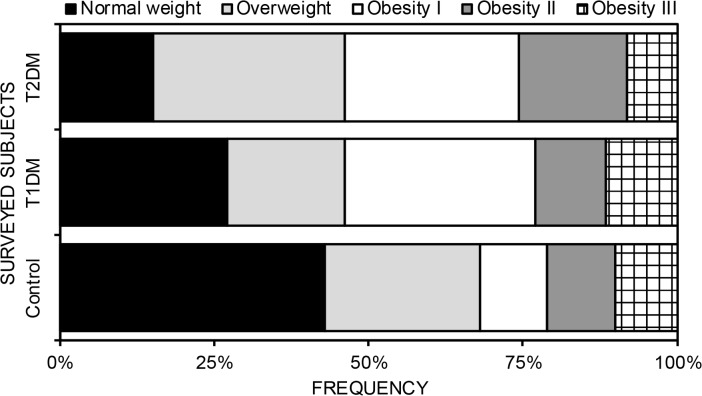
Distribution of diabetic and control subjects according to the different classes of BMI (T1DM: Type 1 Diabetes Mellitus, T2DM: Type 2 Diabetes Mellitus)

The percentages obtained for the different classes of BMI are represented in Fig. 1. In diabetic subjects, regardless of the type of diabetes, obesity was significantly (unpaired *t*-test: *P =* 0.001) more frequent (T1DM: 53.77%, T2DM: 53.92%) and different from controls, where rate of normal-weight subjects (43%) was more important compared to overweighed persons (25%) and the obese (32%). Thus diabetics were significantly more obese than control subjects (Fig. 2). Subjects with T2DM were significantly (unpaired t-test: *P*<0.001) obese and overweight compared to patients with T1DM.

The study of correlation revealed a significant positive relationship between blood glucose and BMI in T1DM subjects (*r* = 0.013, *P*=0.002) and T2DM (*r* = 0.114, *P*=0.001), regardless of gender. The same relationship was observed within control subjects (*r* = 0.331, *P*=0.001).

### State of personal health

The microangiopathies include nephropathy, retinopathy and diabetic neuropathy. Taking into consideration the type of diabetes, diabetic patients with type 1 and 2 have significantly more personal history with regards these diseases than control subjects (Table 2).

**Table 2:** Distribution of the surveyed population according to personal health status

***Parameters ***	***Control subjects***	***Patients with T1DM***		***Patients with T2DM***		***T1DM+T2DM***	
	***N (%)***	***N (%)***	***P***	***N (%)***	***P***	***N (%)***	***P***
Hypertension (HTN)							
Men	08 (21.05)	01 (14.29)	0.569	15 (65.22)	0.002	16 (53.33)	0.013
Women	13 (20.97)	09 (47.37)	<0.001	31 (60.78)	<0.001	40 (57.14)	<0.001
Both genders	21 (21.00)	10 (38.50)	0.006	46 (62.16)	<0.001	56 (56.00)	<0.001
Microangiopathies							
Men	03 (7.89)	02 (28.57)	0.318	04 (17.39)	0.31	06 (36.67)	0.168
Women	02 (3.23)	09 (47.37)	<0.001	15 (29.41)	0.002	24 (34.29)	<0.001
Both genders	05 (5.00)	11 (42.30)	<0.001	24 (32.43)	<0.001	35 (35.00)	<0.001

(N: number of subjects with the pathology, *P*: *P*-value obtained from the Chi-square test)

Hypertension (HTN) was significantly (*P*=0.003) more common in T2DM subjects for both genders combined. The results of the *χ*^2^ test indicate that it is microangiopathies were more common in T1DM particularly in female gender (Table 2).

### Biochemical parameters

**Fig. 3:**
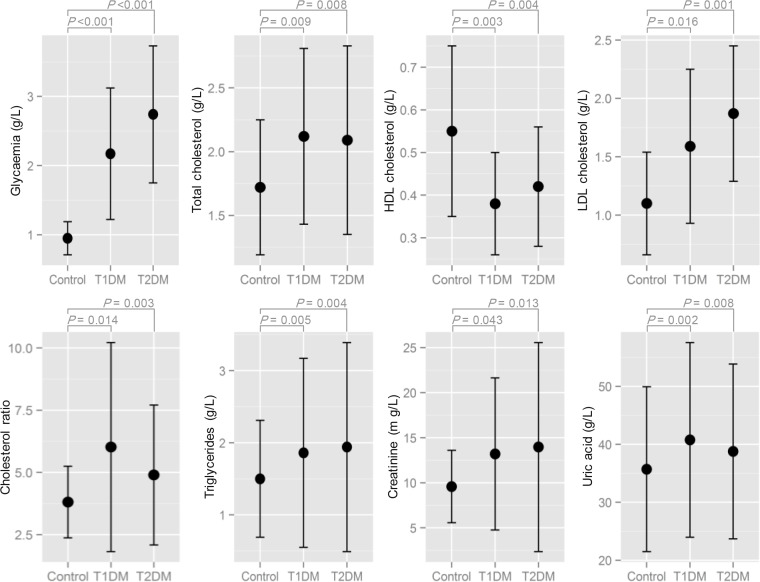
Mean values (black dots) of the assayed biochemical parameters for control subjects and patients with T1DM and T2DM in the area of Tebessa (Northeast Algeria). Vertical bars represent the standard deviation, *P*: *P*-value obtained from unpaired *t*-test

All the studied biochemical parameters were significantly higher in diabetics of both types than in control subjects, except for HDL cholesterol that was significantly higher in control subjects (Fig. 3). By taking into consideration the type of diabetes, there was no significant difference between subjects with T1DM and T2DM. Furthermore, the correlation test applied between blood glucose and various biochemical parameters showed a significant positive correlation with all the assayed biochemical parameters (Table 3).

**Table 3:** Pearson correlation tests between blood glucose and various biochemical parameters measured in the entire study population

***Parameters***	***Control subjects***		***Patients with T1DM***		***Patients with T2DM***	
	***r***	***P***	***r***	***P***	***r***	***P***
Glycaemia vs. total cholesterol	0.520	<0.001	0.450	<0.001	0.250	0.018
Glycaemia vs. HDL cholesterol	0.433	0.048	0.322	0.001	0.120	0.029
Glycaemia vs. LDL cholesterol	0.637	<0.001	0.241	0.003	0.331	0.040
Glycaemia vs. triglycerides	0.170	0.001	0.515	<0.001	0.490	0.001
Glycaemia vs. creatinine	0.250	0.012	0.709	<0.001	0.501	<0.001
Glycaemia vs. uric acid	0.143	0.025	0.234	0.004	0.165	0.021

(*r*: Pearson’s correlation coefficient, *P*: *P*-value)

## Discussion

The age of the surveyed subjects presents records of the same order as in numerous studies, in which the average age of diabetes was 54.7 ± 14 yr regardless of sex and type of diabetes (18). The T2DM subjects of the current study were significantly older than subjects with T1DM. Basically the same findings regarding T2DM have been found (19) an average age of 57 yr; while in 2009 (8) noted an age of 53 ± 10 yr for a 30-diabetic patients in Casablanca (Morocco).

By taking into consideration the type of diabetes, the age is significantly positively correlated with glycaemia. These findings are consistent with other studies such as the National Health Survey conducted in 2005 by the National Institute of Public Health of Algeria, which revealed that the prevalence of total diabetes in Algeria and glycaemia increased significantly with age especially between 35 and 70 yr for patients with T2DM (2). Indeed, the prevalence of diabetes also increases with age, because of the positive association between age and blood glucose (20). In 1999 (12) demonstrated an increase of blood glucose with aging in adult diabetic subjects.

Regardless of patient gender, the prevalence of diabetes increases with age (21). The risk of developing diabetes, especially T2DM, greatly increases with age, where the most affected age class is that of 40–59 yr (20). Indeed, there is a decrease in insulin secretion and increased insulin resistance in elderly patients (22).

In the study population, women significantly outnumber in both groups of diabetes (T1DM: 73.07%, T2DM: 68.91%). These results are similar to other studies in Algeria. Indeed, the total prevalence of diabetes was 14.2% in Tlemcen (Northwest Algeria), where it was higher in women (20.4%) against 10.7% in men (2).

A survey on the lipid profile in diabetics in Morocco indicates that the prevalence of diabetes is higher among women (23). Cicolella (24) have also found similar trends between genders. In fact, the distribution of diabetes by gender reveals globally a female predominance (55%), probably related to the high longevity of women in North Africa and other regions worldwide (25). These gender patterns of diabetes may be related to the social status of women in Muslim societies in general and Algeria or North Africa so far in particular, where women are in the majority of cases, housewives, which means that they exert little effort compared to men (26). This also explains their higher state of obesity compared to men. Moreover, women who presented with diabetes during pregnancy (gestational diabetes) are at higher risk of T2DM (21). Beyond 65 yr, diabetes affects further women (12, 27).

The findings of this study indicated that diabetic subjects of both genders are significantly more obese compared to control subjects. Regarding the type of diabetes, subjects with T2DM are significantly more obese and overweight than subjects with T1DM. These results are similar to those obtained (28) that the prevalence of overweight and obesity in adults over 18 yr is 31.9% and 14.5%, respectively. There are 3 times more T2DM in overweight and 7 times higher in obese (28).

About 25% increase in the prevalence of diabetes in the USA is due to the increasing number of obese (29). In fact, two-thirds of adults with T2DM are overweight. In 80% of cases, diabetes is related to overweight or obesity. Besides the sedentariness and decreased physical activity, obesity plays a major role through insulin resistance (7, 30).

The obesity is characterized by a chronic condition in which the adipose tissue cannot store so normal triglycerides, which resulted in the deposition of these lipids in compartments other than those conferred to this function, as visceral adipose tissue, muscles, liver, heart and pancreas (9, 30–32). Moreover, the visceral adipose tissue releases a large amount of free fatty acids, which promotes hepatic triglyceride synthesis and stimulates hepatic gluconeogenesis and therefore the increase in blood glucose (33).

In addition, the present study revealed that glycaemia was significantly correlated with BMI in both types of diabetes regardless of gender. Obesity and T2DM are closely related in human. Both have a strong genetic component and are associated with insulin resistance (33). Indeed, the risk of diabetes increases linearly with BMI: 2% in overweight subjects, 21% obese. Comparable findings were reported the relationship between BMI and diabetes (34).

The importance of diabetes as a risk factor for a number of diseases including hypertension and microangiopathies has been reported by many studies worldwide (35,36). In our study, diabetic subjects have significantly more diseases including hypertension (HTN) and microangiopathies than control subjects. Regarding hypertension, in 2006 (37) demonstrated a link between diabetes and hypertension, where HTN is readily associated with a constellation of metabolic abnormalities gathered under the term “syndrome X” that includes carbohydrate intolerance or non-insulin dependent diabetes (T2DM), hyperinsulinemia, hypertriglyceridemia and reduced HDL cholesterol. The same results were found by Bonnet & Lavile (38). Hypertension is particularly more frequent and severe in T2DM subjects (39).

The Association HTN–T2DM is particularly common in elderly patients, and is responsible for an increase in cardiovascular risk and an acceleration of the degenerative disease of diabetes. The population of diabetic hypertensive patients is evidently exposed to cardiovascular complications (27, 37). Among the studied 309 patients with T1DM, 120 had at least one microangiopathic complication (35). The care of all elderly diabetics requires setting therapeutic objectives, including glycaemia-based adapted to the patient state. In young diabetics, glycemic lowering reduces the risk of micro– and macroangiopathic complications (35).

Moreover, the studied biochemical parameters were significantly higher in diabetic subjects; except for HDL cholesterol was significantly low and strongly correlated with glucose. In fact, diabetes mellitus is defined as a metabolic disorder, of various etiologies, characterized by the presence of chronic hyperglycemia accompanied by metabolism disruption of carbohydrate, lipid and protein, resulting from a defect in insulin secretion, its activity, or both causes (40).

Insulin exerts a different action on metabolism of carbohydrate, lipid and protein. It stimulates the use of glucose by the liver and its storage as glycogen, whereas adipose tissue increases the up-take and metabolism of glucose by adipocytes, and in muscle, it activates glucose uptake by the cell and glycogen synthesis (30,32,33,40).

Plasma lipids are purified by the action of lipoprotein lipase which tissue synthesis requires the presence of insulin. The latter stimulates lipogenesis and inhibits lipolysis in adipose tissue and liver. In addition, insulin decreases the rate of circulating amino acid by increasing the cellular uptake of amino acids; by increasing protein synthesis, the latter is achieved by stimulation of the activation of amino acids and mRNA ribosomal reading, but also by decreasing the proteolysis (28). Therefore, the absence of insulin (T1DM) or insulin resistance (T2DM) induces the following consequences:

– Increases the rate of glucose in the blood, main responsible for the observed hyperglycemia.
– Increases the lipoprotein (total cholesterol, LDL cholesterol, triglycerides) with a decrease in HDL cholesterol in the blood, indicating a dyslipidemia.
– Increases the rate of proteins in the blood (creatinine and uric acid), characteristic of renal failure.

## Conclusion

This study confirms the relationship between diabetes, particularly T2DM, and many complications highlighted by several studies. Disturbances recorded in the results of biochemical tests are observations that should be noted but that need further investigation to better illustrate their connections with diabetes. Thus, conducting studies a larger scale (national or regional) is required to better identify the magnitude of the problem, particularly in developing countries. Countries and national organizations urgently need to invest more in the diabetes control in particular T2DM. The current survey is a first approach to know the link between diabetes and some biochemical parameters. Further of larger studies are surely needed to better understand diabetes and its associated pathologies.

## Ethical considerations

Ethical issues (Including plagiarism, informed consent, misconduct, data fabrication and/or falsification, double publication and/or submission, redundancy, etc.) have been completely observed by the authors.
